# Role of PCPs in diagnosing dementia in traditional Medicare and Medicare Advantage

**DOI:** 10.1002/alz.14559

**Published:** 2025-10-09

**Authors:** Sidra Haye, Mireille Jacobson, Julie Zissimopoulos

**Affiliations:** ^1^ USC Price School of Public Policy University of Southern California, Ralph and Goldy Lewis Hall Los Angeles California USA; ^2^ Leonard D. Schaeffer Institute for Public Policy & Government University of Southern California, Way, Verna & Peter Dauterive Hall Los Angeles California USA; ^3^ Leonard Davis School of Gerontology University of Southern California Los Angeles California USA

**Keywords:** Alzheimer's disease, dementia diagnosis, Medicare, Medicare Advantage, primary care

## Abstract

**INTRODUCTION:**

This paper quantifies how incident dementia diagnosis rates vary for similar beneficiaries in traditional Medicare (TM) and Medicare Advantage (MA) seen by the same primary care provider (PCP).

**METHODS:**

This cohort analysis used 2016 to 2018 data for Medicare beneficiaries. Using a propensity score‐matched sample of beneficiaries with similar likelihood of MA enrollment, we estimated linear probability models with PCP fixed effects of incident dementia diagnosis in 2017 for beneficiaries in MA relative to beneficiaries in TM.

**RESULTS:**

Among a matched sample of 15,410,030 beneficiaries, accounting for both provider and patient characteristics, the incident dementia diagnosis rate was 0.11 percentage points lower for MA beneficiaries compared to TM beneficiaries attributed to the same PCP. MA patients were less likely to be seen by dementia specialists.

**DISCUSSION:**

Differences in system‐level factors such as access to dementia specialists is a contributing factor to differences in diagnosis rates in MA and TM.

**Highlights:**

- In this study, we quantify how incident dementia diagnosis rates vary for similar beneficiaries in TM and MA seen by the same PCP.
- To investigate the role of insurance design on dementia diagnosis, we examined differences in diagnosis rates for TM and MA beneficiaries seen by the same PCP.
- Among a matched sample of TM and MA beneficiaries with assigned PCPs in 2017, incident dementia diagnosis rates were 0.11 percentage points lower for MA beneficiaries compared to TM beneficiaries seen by the same PCP.
- MA patients were less likely to be seen by dementia specialists compared to TM beneficiaries seen by the same PCP.

## 1 BACKGROUND

With an aging population, the number of older Americans with Alzheimer's disease and related dementias (ADRD) is expected to reach 13 million by 2050.^1^ Between 20% and 50% of persons with dementia and an even higher number of persons with mild cognitive impairment are undiagnosed. ^2^, ^3^, ^4^, ^5^ A timely diagnosis can help millions of Americans living with dementia plan for future financial and caregiving needs.^1^, ^5^, ^6^

Importantly, with the approval of new medications for individuals at the early stages of dementia, a timely diagnosis can also facilitate access to potentially disease‐modifying treatments.^7^ Primary care providers (PCPs) are central to the dementia diagnosing process as they make the majority of initial diagnoses and provide most post‐diagnosis care.^8^, ^9^ Frequent contact and long‐term relationships with patients allow PCPs to detect the early signs of cognitive impairment.^10^, ^11^ Many PCPs believe that they are at the front line of dementia care and report regularly answering questions related to dementia.^11^ Policies such as the Patient Protection and Affordable Care Act of 2010 also recommend greater involvement of PCPs in early detection of cognitive impairment through the annual wellness visits (AWVs).^12^ Centers of Medicare and Medicaid requires, among other things, that providers detect cognitive impairment during these comprehensive primary care visits.^12^ However, many studies suggest that PCPs face many challenges in diagnosing dementia, including lack of training and time and a shortage of dementia specialists to refer patients.^3^, ^9^, ^11^ Insurance design and other healthcare system factors may create additional challenges for PCPs in the diagnosing process, such as imposing time constraints on patient visits and/or limiting referral networks or diagnostic services.

More than half of all Medicare beneficiaries now choose to receive their health benefits through private Medicare Advantage (MA) plans. ^13^ Unlike traditional Medicare (TM), MA plans receive capitated payments per beneficiary per month from the federal government to manage the cost of and care for their enrollees. Most MA plans offer reduced cost‐sharing and supplemental benefits that are particularly attractive to traditionally high‐need Medicare beneficiaries such as racial and ethnic minority groups and dual‐eligible beneficiaries. ^14^ However, these extra benefits typically come at the cost of narrow provider networks and prior authorization requirements,^15^, ^16^ which create challenges for dementia diagnosis.

To investigate the role of insurance design on dementia diagnosis, we examined differences in diagnosis rates for TM and MA beneficiaries seen by the same PCP. Analyzing patients seen by the same PCP allows us to hold constant a provider's general diagnostic skill or propensity to diagnose. Accounting for both provider and patient characteristics enables us to isolate differences in diagnosis that stem from the unique features of MA relative to TM, such as narrow networks and restricted access to specialists. To shed further light on this interpretation, we also examined whether actions related to dementia diagnosis, including the probability of seeing a dementia specialist and of having an AWV, differed for TM and MA beneficiaries who are seen by the same PCP.

**RESEARCH IN CONTEXT**

1. **Systematic review**: Dementia diagnosis and care differ for beneficiaries in MA and TM for multiple reasons, including differences in the characteristics of enrollees, the providers they see, and in the insurance design of the healthcare systems.
2. **Interpretation**: Accounting for both differences in beneficiary characteristics and providers does not fully explain the differences in diagnosis rates among beneficiaries in MA and TM. Differences in system‐level factors such as access to dementia specialists are a contributing factor to differences in diagnosis rates among beneficiaries in MA and TM.
3. **Future directions**: A critical next step is to examine how changes in payments to MA plans impact, within provider differences, the diagnosis rates for TM and MA beneficiaries.

## 2 METHODS

### 2.1 Data and sample

Our primary data sources were 100% of TM claims and MA encounter data for all Medicare beneficiaries. Our sample included Medicare beneficiaries 65 years and older who were continuously enrolled in Medicare Part D and either TM Part A and B or MA in 2016 through 2018 or died in 2017 or 2018. We used a rigorous, previously published algorithm to identify dementia diagnosis using claims and encounter data that verify a diagnosis in any healthcare setting with a second diagnosis, a dementia symptom, or a dementia drug claim.^2^, ^17^ In our prior work we assessed the quality of the MA encounter data for examining dementia diagnosis for MA beneficiaries. We used Optum's deidentified Clinformatics Data Mart (CDM) database for the years 2015 to 2018 to assess the quality of dementia diagnosis found in MA encounter data. CDM data include all claims for patients who are provided health insurance coverage by a large insurance company and include procedure and diagnosis codes and dates of service. The CDM data are collected directly from the providers within the insurance company. We used claims for MA enrollees from the same insurer in the CDM and in the MA encounter data. We validated the quality of the MA encounter files by comparing rates of diagnosed dementia from the CDM data to the MA encounter data from the same large insurance company and found that the data provided reliable estimates of diagnosed dementia among MA beneficiaries.^2^ To identify individuals with an incident dementia diagnosis, we required that the at‐risk population have no dementia diagnosis 1 year before the year of interest, that is, no dementia diagnosis in 2016.

Our goal was to examine differences in diagnosis rates for MA and TM beneficiaries holding constant beneficiary and PCP‐level factors. Thus, we excluded from our sample beneficiaries who had no primary care visits in 2017. We identified PCPs as providers in the specialty of general practice, internal medicine, family medicine, nurse practitioners, physician assistants, and clinical nurse specialists based on their National Provider Identifier (NPI) in the National Plan and Provider Enumeration System (NPPES) database. For each beneficiary, we identified their PCP as the provider with whom they had the maximum number of office visits (includes evaluation and management visits) among all the PCPs they saw in 2017. In case of a tie, beneficiaries were assigned to the PCP of the first primary care visit in 2017.^16^, ^18^

We used propensity score matching to match TM and MA beneficiaries on their probability of enrolling in MA based on their observable characteristics including gender, race, 5‐year age group, dual eligibility status, chronic conditions (acute myocardial infarction [AMI], atrial fibrillation, hypertension, hyperlipidemia, diabetes, stroke), county education, and income. We matched using nearest‐neighbor matching with a width of 0.1 of the standard deviation of the logit of the propensity score. This method selects a TM beneficiary whose propensity score best matches the propensity score of a MA beneficiary for enrolling in MA. Each TM beneficiary is matched to a unique MA beneficiary. Table S1 in the supplementary material describes the sample construction.

### 2.2 Outcomes

Our main outcome of interest was incident dementia diagnosis in 2017 among beneficiaries without any dementia diagnosis in 2016. We identified dementia diagnosis codes (Table S2) from inpatient, outpatient, home health, carrier, and skilled nursing facility claims. We used dementia diagnosis codes, verified with a second dementia diagnosis, or Part D claims for treatment for dementia symptoms or claims for dementia symptoms to exclude potential rule‐out diagnoses.

As differences in insurance design likely impact actions associated with a dementia diagnosis,^8^, ^10^ we additionally examined differences in the probability of seeing a dementia specialist and of receiving an AWV. We identified dementia specialists as physicians in the specialty of neurology, psychiatry, neuropsychiatry, geriatrics, and geriatric psychiatry.^8^ Individuals who had at least one visit in 2017 with physicians in these specialties were identified as having seen a dementia specialist. We used Healthcare Common Procedure Coding System (HCPCS) codes G0428 and G0439 to identify AWVs. AWV was examined because cognitive assessment is a required component of the visit.

### 2.3 Covariates

Patient characteristics, including patient age, sex, dual eligibility race, and ethnicity, were obtained from the Master Beneficiary Summary Files (MBSFs). We used the MBSF's Research Triangle Institute race variable to identify beneficiary race and ethnicity. We obtained county characteristics including high‐school graduation rate and mean income for older adults from the American Community Survey (ACS). Chronic conditions for TM and MA beneficiaries were identified using the Chronic Conditions Warehouse (CCW) algorithms. We determined enrollment in health maintenance organization (HMO) and preferred provider organization (PPO) plans for MA beneficiaries using MBSFs. MA beneficiaries with a Plan Type Code variable value of 001 were classified as in HMO plans, and those with a value of 004 were classified as in PPO plans.

### 2.4 Statistical analysis

We first compared rates of incident dementia diagnosis in TM and MA using the full sample of beneficiaries to whom we could attribute a PCP. Using the matched sample, we then estimated linear probability models to examine the differences in dementia diagnosis rates in MA compared to TM adjusting for beneficiary characteristics. The dependent variable was a binary variable that equaled 1 if the beneficiary received an incident dementia diagnosis in 2017 and zero otherwise. The key explanatory variable was a binary variable that equaled 1 if the beneficiary was enrolled in a MA plan in that year and zero if the beneficiary was in TM. The main specification controlled for beneficiary demographics (age, sex, race and ethnicity, dual eligibility status), CCW chronic conditions, and county characteristics (high‐school graduation rate and income levels for older adults).

We then added PCP fixed effects (FEs) and compared the estimate from the main specification. Models with PCP FEs allowed us to examine the mean differences in diagnosis rates between TM and MA beneficiaries seen by the same PCP. As some of the differences in diagnosis rates between MA and TM may be due to differences in PCPs of beneficiaries in MA and TM including but not limited to a PCP's patient population, dementia diagnostic training, or general diagnostic practices, we hypothesized that including PCP FEs would reduce the estimated difference in dementia diagnosis rates in MA relative to TM. To further examine the role of system‐level factors such as provider networks, we separately compared diagnosis rates for TM beneficiaries with those for MA beneficiaries in HMO plans, which have narrow networks, and PPO plans, which have broader networks.

We used the same basic linear probability models to analyze the probability of seeing a dementia specialist and the probability of an AWV visit. Robust standard errors are reported in the main text. We performed numerous sensitivity checks, including limiting MA encounter data plans with complete contracts to address potential issues with data completeness in the encounter data,^19^ using inverse probability‐weighted linear regressions as an alternative approach to matching, matching beneficiaries on the Charlson Comorbidity Index, and restricting our analysis to providers with at least 25 patients each in TM and MA to ensure the providers treated adequate numbers of patients of each type. To control for differences in visits that may differ based on MA versus TM access and benefit design, we estimated models that controlled for the number of PCP visits. We tested the sensitivity of our estimates when excluding incident dementia diagnoses based on chart reviews, which are common in MA and may capture coincident diagnoses. ^20^, ^21^ As differences in diagnosis might be due to no dementia diagnosis or lack of a second diagnosis, we examined differences in diagnosis rates where the dependent variable was a single dementia diagnosis in 2017. Finally, as the FEs can be estimated only for PCPs who have at least one patient with an incident dementia diagnosis, we limited our analyses to these PCPs. All analyses were performed using SAS Enterprise Guide 7.1.

## 3 RESULTS

### 3.1 Beneficiary characteristics

Table 1 presents summary statistics for MA and TM before and after propensity score matching. From an overall sample of 19,290,942 beneficiaries, the matching procedure resulted in an analytic sample of 15,410,030. Our matched sample consisted of 7,705,015 MA beneficiaries (mean [SD] age, 75.80 [6.71] years; 58.26% female, 80.62% White, 7.53% Black, 5.92% Hispanic, and 3.55% Asians) and 7,705,015 TM beneficiaries (mean [SD] age,75.89 years [6.84], 58.55% female, 80.77% White, 7.62% Black, 5.55% Hispanic, and 3.62% Asians). Unlike the unmatched sample, the matched sample looked similar based on demographic characteristics, most notably based on beneficiary sex and race. Beneficiaries with an incident ADRD dementia diagnosis represent 2.80% of the MA beneficiaries and 3.14% of TM beneficiaries in our matched sample. Roughly 85.6% (205,639) of PCPs saw both TM and MA beneficiaries and accounted for 14,194,729 patients.

**TABLE 1:** Characteristics of beneficiaries in the study sample.

	Unmatched		Matched	
	TM	MA	TM	MA
Incident dementia	3.24%	2.76%	3.14%	2.80%
Female	60.09%	58.45%	58.55%	58.25%
White	85.03%	73.10%	80.77%	80.62%
Black	5.59%	9.47%	7.62%	7.53%
Asian	3.99%	11.49%	5.55%	5.92%
Hispanic	2.85%	3.77%	3.62%	3.55%
AIAN	0.30%	0.15%	0.16%	0.16%
Other	2.24%	2.01%	2.28%	2.22%
Mean age	76.01	75.67	75.89	75.80
Age 65 to 69	19.17%	19.14%	19.07%	18.72%
Age 70 to 74	23.19%	24.55%	23.87%	24.33%
Age 75 to 79	22.44%	22.90%	22.64%	22.88%
Age 80 to 84	14.25%	14.08%	14.41%	14.27%
Age 85 to 89	8.63%	7.90%	8.47%	8.19%
Age 90+	5.11%	3.97%	4.48%	4.21%
Dual eligible	12.07%	12.35%	12.03%	11.79%
Acute Myocardial Infarction	0.86%	0.77%	0.82%	0.81%
Atrial fibrillation	11.18%	9.55%	10.84%	10.23%
Hypertension	69.76%	70.27%	70.24%	69.21%
Hyperlipidemia	61.25%	63.10%	62.33%	62.26%
Diabetes	30.48%	33.44%	32.24%	31.62%
Stroke	3.90%	3.22%	3.58%	3.41%
*N*	10,776,719	8,514,223	7,705,015	7,705,015

*Note*: This table presents summary statistics for Traditional Medicare (TM) and Medicare Advantage (MA) beneficiaries with assigned primary care providers (PCPs) in 2017. The first two columns present summary statistics for unmatched samples. The last two columns present summary statistics for the matched sample. Propensity score matching was done where beneficiaries were matched on female, race, age, chronic conditions, and county characteristics.

### 3.2 Differences in dementia diagnosis rates in TM and MA seen by the same PCP

**FIGURE 1:**
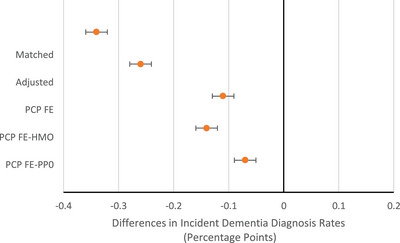
Differences in dementia diagnosis rates in MA versus TM without and with PCP fixed effects (FEs). The matched specification compares incident dementia diagnosis rates in MA to TM in 2017 for the matched sample (*N* = 15,410,030) The adjusted specification controls for beneficiary demographics, chronic conditions, and county characteristics using a linear regression model (*N* = 15,410,030). The PCP FE specification includes a PCP FE to compare differences in diagnosis rates within PCPs (*N* = 15,410,030). The PCP FE‐HMO specification compares within‐PCP differences in diagnosis rates for MA beneficiaries in HMO plans compared to TM (*N* = 13,331,964). The PCP FE‐PPO specification does the same for MA beneficiaries in PPO plans (*N* = 11,232,851).

Figure 1 presents the differences in incident dementia diagnosis rates between TM and MA in the matched sample. The first specification presents the difference in diagnosis rates in MA compared to TM. In a matched sample, the incident dementia diagnosis rate was 0.34 percentage points (SD 0.01) lower in MA compared to TM. The second specification adjusted for demographics, chronic conditions, and county characteristics of beneficiaries using a linear regression model. After adjusting for beneficiary characteristics, the estimated difference in dementia diagnosis rates in MA compared to TM fell to 0.26 (SD 0.01) percentage points. The third specification shows the estimates after we included PCP FEs. The incident dementia diagnosis rate in MA remained 0.11 (SD 0.01) percentage points lower than in TM for beneficiaries seen by the same PCP. The fourth and fifth specifications present the adjusted difference in diagnosis rates between MA beneficiaries in HMO plans and then in PPO plans compared to TM beneficiaries seen by the same PCP. Diagnosis rates are 0.14 percentage points (SD 0.01) lower for MA beneficiaries in HMOs compared to TM but only 0.07 percentage points (SD 0.01) lower for MA beneficiaries in PPOs compared to TM (Table S3 shows estimates for the unmatched sample).

In sensitivity analyses (Table S4), we first limited our analyses to MA plans with complete contracts, as identified by prior work, to address concerns about reporting issues in MA encounter data.^20^ Limiting the analysis to complete contracts had no meaningful impact on our estimates for within‐PCP differences in incident dementia diagnosis rates. Second, as an alternative to matching, we estimated inverse probability‐weighted linear regressions, where the individual probability of enrolling in MA was used as weight, and found similar results (Table S5). Third, to further control for differences in beneficiary health status, we also matched TM and MA beneficiaries on Charlson Comorbidity Index in addition to the aforementioned covariates. The estimate from this analysis was slightly larger (−0.19 vs 0.11). Fourth, we examined the robustness of our results to limiting our analyses to PCPs that saw at least 25 patients each in TM and MA, indicating they saw substantial numbers of each type of patient. The estimate was qualitatively similar albeit slightly smaller in magnitude with this restriction (−0.08 vs −0.11). Fifth, we controlled for the number of visits patients had with their assigned PCPs in 2017, since this may differ in MA and TM in ways that can impact the probability of diagnosis independent of physician practice patterns. Controlling for the number of visits with the PCP, within‐PCP differences in diagnosis rates in MA and TM were larger. Sixth, we examined the differences in diagnosis rates after excluding incident dementia diagnoses from chart reviews. While dementia diagnoses were not used to risk‐adjust payments to MA plans in 2017, prior work suggested that MA plans used chart reviews to increase their payments. ^20^, ^22^ The estimated difference in diagnosis rates was much larger after excluding the incident dementia diagnoses from the chart reviews (−0.53 vs −0.11). Seventh, we examined within‐PCP differences in incident dementia diagnoses for beneficiaries with no dementia diagnosis in 2016 and at least one dementia diagnosis in 2017. The estimated difference was slightly larger for this sample (−0.18 vs −0.11). Finally, as FEs can only be estimated for PCPs with variation in diagnosis rates, we limited the sample to PCPs who saw at least one patient with incident dementia diagnosis in 2017 and found similar results.

### 3.3 Differences in probability of seeing a dementia specialist and in receipt of AWVs for TM and MA beneficiaries seen by the same PCP

Within a PCP, MA beneficiaries were 0.79 percentage points less likely to see a dementia specialist (Table 2). The probability of seeing a dementia specialist was 0.82 percentage points lower for MA beneficiaries in HMO plans compared to TM and 0.63 percentage points lower for MA beneficiaries in PPO plans compared to TM. In contrast to TM beneficiaries seen by the same PCP, MA beneficiaries had a 3.78‐percentage‐point higher probability of receiving an AWV. MA beneficiaries in both HM0 and PPO plans had higher rates of AWV compared to TM beneficiaries.

**TABLE 2:** Within‐PCP differences in probability of seeing a dementia specialist and receiving an annual wellness visit.

	Probability of seeing a dementia specialist			Probability of receiving an AWV		
	MA versus TM	MA‐HMO versus TM	MA‐PPO versus TM	MA versus TM	MA‐HMO versus TM	MA‐PPO versus TM
MA	−0.79***	−0.82 ***	−0.63***	3.78***	4.02***	2.62***
	(0.01)	(0.01)	(0.01)	(0.02)	0.03	0.03
*N*	15,410,030	13,331,964	11,232,851	15,410,030	13,331,964	11,232,851
Mean TM	3.94%			37.4%		

*Note*: The first column presents the adjusted difference in probability of seeing a dementia specialist for MA versus TM for patients attributed to the same PCP. The second and third columns present the same differences for MA‐HMO compared to TM and MA‐PPO compared to TM. Differences in the probability of annual wellness visits (AWVs) are examined in columns 4 to 6.

## 4 DISCUSSION

Many patients have frequent contact and maintain long‐term relationships with their PCPs.^23^ Consequently, these providers are at the forefront of diagnosing dementia and make the majority of initial dementia diagnoses (Table S6).^10^, ^11^ PCPs conduct cognitive assessments, refer patients to specialists, manage coexisting chronic conditions, prescribe medications, and help decide on goals of care. With an aging population and shortage of dementia specialists, PCPs’ role in dementia diagnosis and care is likely to become even more important.^1^, ^9^ To our knowledge, this is the first study to examine the differences in dementia diagnosis rates among TM and MA beneficiaries seen by the same PCP. In this work, we estimated differences in dementia diagnosis in MA relative to TM adjusting for both observable differences in beneficiary characteristics in TM and MA (using propensity score matching and regression analyses) and provider selection by focusing on MA and TM beneficiaries seen by the same PCP.

At baseline, in an unmatched sample, the incident dementia diagnosis rate was 0.48 percentage points lower in MA compared to TM. Controlling for demographic characteristics and chronic conditions using matching and regression analyses, the difference in dementia diagnosis rates in MA compared to TM decreased to 0.26 percentage points. Including PCP FEs further reduced the difference in diagnosis rates to 0.11 percentage points, suggesting that at least half of the difference in diagnosis rates was due to differences in patient characteristics and provider selection.

However, approximately one‐fourth of the difference in diagnosis rates in TM and MA remained even among beneficiaries seen by the same PCP. Our results suggest that incident dementia diagnosis rates are around 3.5% lower for MA beneficiaries compared to TM beneficiaries seen by the same PCP. While some of this difference may still reflect differences in patient characteristics, other analyses suggest the important role of insurance design. Relative to TM, within‐PCP differences in diagnosis rates were larger for MA beneficiaries in HMO plans and much smaller for MA beneficiaries in PPO plans. In contrast to PPO plans, HMO plans typically have more restrictive provider networks and do not cover out‐of‐network visits.^24^ As many PCPs report being uncomfortable making a dementia diagnosis^11^ and many refer patients with suspected cognitive impairment to specialists,^25^ these results suggest that some of the differences in diagnosis rates might be due to differential access to dementia specialists. Consistent with this, we found that, compared to TM beneficiaries, MA beneficiaries seen by the same PCP were about 20% less likely to see a dementia specialist. We also found that, compared to TM beneficiaries, the probability of seeing a dementia specialist was 21% lower for MA beneficiaries in HMO plans and 16% lower for MA beneficiaries in PPO plans.

Consistent with prior work that found self‐reported AWV rates were overall higher in MA compared to TM,^12^ we found that MA beneficiaries seen by the same PCP as TM beneficiaries had higher rates of AWV. Higher rates of AWV and lower rates of dementia diagnosis are consistent with lower rates of cognitive assessments at these visits.^12^ As dementia diagnosis was not used to risk‐adjust payments to MA plans prior to 2020,^26^ this may also reflect the role of AWVs in escalating coding intensity of only diagnoses that impact MA plan payments.^27^

This study has several limitations. First, it was an observational study and controlled for observable differences in TM and MA beneficiaries but could not fully account for unobservable differences in patient characteristics. While matching and regression analyses can decrease the bias due to selective enrollment in MA, it is unlikely to completely correct for selection. Second, the goal of this study was to examine the differences in diagnosed dementia rates among TM and MA beneficiaries seen by the same PCP. These differences may not generalize to persons who do not have an assigned PCP. Third, the MA encounter files are relatively new, and data completeness varies across MA plans.^28^ While prior work found that MA encounter data provided reliable estimates of diagnosed dementia^2^ and restricting our analyses to complete contracts yielded similar results, incomplete data can impact our sample and estimates. Fourth, by the nature of the method, including PCP FEs excludes PCPs without variation in the dependent variable, that is, PCPs without any patients with incident dementia diagnosis. Thus, our results may not generalize to these PCPs.

This observational study used 100% of data for MA and TM beneficiaries to examine differences in diagnosed dementia rates in a primary care setting. We found that, even after adjusting for patient characteristics, incident dementia diagnosis rates were significantly lower for MA beneficiaries compared to TM beneficiaries seen by the same PCP. Additionally, dementia specialist visits and AWV receipt varied among patients seen by the same PCP based on enrollment in MA compared to TM. Our findings suggest limited access to dementia specialists for MA beneficiaries may play an important role in explaining the differences in diagnosis rates. Future work should examine the impact of changes in incentives to code for dementia on within‐provider differences in diagnosing rates.

## CONFLICT OF INTEREST STATEMENT

The authors declare there is no conflict of interest.

## CONSENT STATEMENT

Consent was not necessary.

## Supporting information



Supporting Information

Supporting Information
