# Supplementary material for: Role of PCPs in diagnosing dementia in traditional Medicare and Medicare Advantage
Source: Alzheimers Dement. 2025 Oct 9;21(10):e14559. doi: 10.1002/alz.14559 (PMC12510135; doi:10.1002/alz.14559)
Supplement: Supplementary file 1 — Supporting Information [file ALZ-21-e14559-s002.docx]

**Supplemental Section**

| **Table S1: Sample Creation** | | |
| --- | --- | --- |
|  | **TM** | **MA** |
| Beneficiaries 65 years and over, continuously enrolled in 2016,2017,2018 | 14,271,750 | 12,869,059 |
| All beneficiaries at risk of dementia in 2017 | 13,217,294 | 12,127,098 |
| Beneficiaries for who we were able to attribute PCPs in 2017 | 10,776,719 | 8,514,223 |
| Matched Sample | 7,705,015 | 7,705,015 |
| Notes: This table provides the details for how the final sample was created. At risk population is defined as individuals with Part D who were enrolled in either TM or MA for the years 2016, 2017 and 2018, and no incident dementia diagnosis in 2016. | | |

| **Table S2: Codes** |
| --- |
| **Dementia Codes:** Dementia is defined using the following codes from the *International Classification of Diseases, Ninth* and *Tenth Revisions*: 331.0, 331.11, 331.19, 331.2, 331.7, 290.0, 290.10, 290.11, 290.12, 290.13, 290.20, 290.21, 290.3, 290.40, 290.41, 290.42, 290.43, 294.0, 294.10, 294.11, 294.20, 294.21, 294.8, 797, 331.82, F01.50, F01.51, F02.80, F02.81, F03.90, F03.91, F05, G13.8, G30.0, G30.1, G30.8, G30.9, G31.01, G31.09, G31.1,G31.2, G31.83, G94, and R41.81. Diagnosis codes are identified in the inpatient, outpatient, home health care, skilled nursing facility, and carrier settings. Part D claims for treatment of dementia symptoms with donepezil, galantamine, rivastigmine, or memantine. Dementia symptoms such as amnesia, aphasia, mild cognitive impairment, and apraxia and agnosia were identified with the following ICD-9 and ICD-10 codes:  780.93, 784.3, 331.83, 784.69, R41.1, R41.2, R41.3, R47.01, R48.1, R48.2, R48.8, and G318.4.  See details  https://github.com/USCSchaefferCenterDataCore/Schaeffer-Dementia-Algorithm |

| **Table S3: Estimates for unmatched sample** | | |
| --- | --- | --- |
| MA | -0.26*** | -0.12*** |
|  | (0.01) | (0.01) |
| Controls | X | X |
| PCP FE |  | X |
| N | 19,234,470 | 19,234,470 |
| Notes: This table presents regression estimates with controls (column 1) and with PCP FE (column 2) using the unmatched sample. | | |

| **Table S4: Complete Contracts** | | | |
| --- | --- | --- | --- |
| Dependent Variable | Incident dementia diagnosis | Probability of seeing a specialist | Probability of an AWV |
| MA | -0.11*** | -0.786*** | 6.81*** |
|  | (0.01) | (0.01) | (0.03) |
| N | 11,974,692 | 11,974,692 | 11,974,692 |
| Notes: This table presents results where MA sample is limited to beneficiaries in MA plans with complete data. The first column examines within PCP differences in diagnoses rates for MA beneficiaries in complete contracts compared to TM beneficiaries. In Column 2 the dependent variable is probability of seeing a dementia specialist. Column 3 shows results where the dependent variable is probability of an annual wellness visit. | | | |

|  | | **Table S5: Sensitivity Analyses** | | | | | |  |
| --- | --- | --- | --- | --- | --- | --- | --- | --- |
|  | IPW | | Matching on CCI | at least 25 patients in TM and in MA each | Controlling for number of visits with assigned PCPs | Excluding Chart Reviews | At least 1 Dementia diagnosis  In 2017 | PCPs with at least 1 incident dementia diagnosis |
| MA | -0.12*** | | -0.19*** | -0.08*** | -0.28*** | -0.53*** | -0.18*** | -0.12*** |
|  | (0.01) | | (0.01) | (0.01) | 0.01 | (0.01) | (0.01) | (0.01) |
| N | 19,234,470 | | 15,491,700 | 9,343,538 | 15,410,030 | 15,379,787 | 16,383,868 | 13,592,736 |
| Notes: Column 1 presents estimates for within PCP differences in diagnoses rates using inverse propensity weighting where propensity of enrolling in MA is used as weights in regression analysis. Column 2 examines within PCP differences in diagnosis rates where beneficiaries are matched on sex, race, age, chronic conditions, county education and income, and Charlson Comorbidity Index Index (CCI). Column 3 presents results where the sample is limited to PCPs that had at least 25 TM beneficiaries and 25 MA beneficiaries attributed to them in 2017. Column 4 controls for the number of visits the beneficiary had with the assigned PCP. Column 5 examines within PCP differences in diagnosis rates for MA excluding chart reviews compared to TM. Column 6 examines within PCP differences where dependent variable is unverified incident dementia diagnosis defined as no diagnosis in 2016 and at least one diagnosis in 2017. Column 7 presents within PCP differences for sample of PCPs what saw at least one patient with an incident dementia diagnosis in 2017. | | | | | | | | |

| **Table S6: Diagnosing Provider** | | |
| --- | --- | --- |
|  | TM | MA |
| PCP makes the incident dementia diagnosis | 50.5% | 54.9% |
| Specialist makes the incident dementia diagnosis | 3.69% | 3.19% |
| PCP makes the verifying diagnosis | 46.9% | 49.99% |
| Specialist makes the verifying diagnosis | 4.1% | 3.83% |
| Notes: This table shows whether incident and verifying dementia diagnosis were made by a PCP or a dementia specialist for TM and MA beneficiaries with an incident dementia diagnosis and assigned PCP. | | |
